# Antibiogram of pathogenic bacterial isolates from various samples in Prince Sultan Hospital, Western Region, Saudi Arabia. Retrospective study from 2020 to 2024

**DOI:** 10.3389/frabi.2026.1872911

**Published:** 2026-08-12

**Authors:** Ebtehal H. Alqurashi, Najla A. Obaid, Alyaa Alzahrani, Fahad S. Alshareef, Najlaa M. Mohandes, Areej M. Althyabi, Sahar Albugami, Sultan Almammari

**Affiliations:** 1 Department of Laboratory, Prince Sultan Forces Hospital, Ministry of Defense, Taif, Saudi Arabia; 2 Department of Pharmaceutical Sciences, College of Pharmacy, Umm Al Qura University, Makkah, Saudi Arabia; 3 Department of Biology, College of Science, Taif University, Taif, Saudi Arabia

**Keywords:** antimicrobial resistance, carbapenem-resistant Enterobacteriaceae, antibigram, healthcare-associated infections, Western Saudi Arabia

## Abstract

**Background:**

Antibiograms support empirical therapy by providing local antimicrobial susceptibility data, guiding clinical decisions, and stewardship efforts. Rising multidrug-resistant (MDR) bacteria highlight the importance of continuous antimicrobial resistance (AMR) surveillance to inform local treatment guidelines.

**Objectives:**

The purpose of this study was to assess AMR rates in clinical pathogenic isolates obtained from community-acquired infections at a general hospital in Western Saudi Arabia.

**Methods:**

Antibiogram data were analyzed retrospectively from January 2020 to September 2024, focusing on pathogenic bacteria and AMR isolates.

**Results and conclusion:**

This study shows high rates of ESBL-producing *E. coli* and the rapid emergence of resistance to last-resort antibiotics, indicating therapeutic exhaustion despite stable resistance to older drug classes. These trends, likely driven by antibiotic overuse during COVID-19, highlight shrinking treatment options and the urgent need to strengthen antimicrobial stewardship and reassess empirical therapies.

## Introduction

The incidence of antimicrobial resistance (AMR) incidence has a particularly severe impact on developing countries, such as Saudi Arabia. Insufficient infection control practices, combined with the misuse of antibiotics, can contribute to the spread of resistant pathogens in overcrowded hospitals (Salam et al., 2023). Healthcare-associated infections (HAIs) and limited resources for comprehensive antimicrobial antibiograms are factors contributing to the spread of AMR (Zaha et al., 2019).

The increasing resistance among Gram-negative organisms, such as *Escherichia coli*, *Pseudomonas aeruginosa*, *Acinetobacter baumannii*, *Salmonella*, *Klebsiella pneumoniae*, and Gram-positive organisms such as *Staphylococcus aureus*, presents a significant public health threat. Increasing antibiotic resistance makes it more difficult to treat serious infections caused by these pathogens, such as bloodstream infections, pneumonia, wound infections, and urinary tract infections (Ahmad et al., 2023; Elfadadny et al., 2024).

In order to combat AMR, antibiograms provide vital local data that can guide empirical therapy. Whenever access to highly effective, costly antibiotics is restricted, antibiograms provide clinicians with evidence-based recommendations for antibiotic use, ensuring that the most appropriate treatment is administered in a timely manner. As an alternative to inappropriate therapy that can exacerbate resistance development, antibiograms play a crucial role (Saleem et al., 2018).

There is an alarmingly high rate of antibiotic resistance in Saudi Arabia, according to several studies. According to a study by Abdallah et al. (2020), 30% of isolates were ESBL-positive, while 74% of the isolates were multidrug resistant. Due to unregulated antibiotic use and inadequate healthcare infrastructure, these resistance trends are often more severe in developing countries (Ehsan, 2025).

Regionally, AMR is well recognized; however, many tertiary care hospitals lack data on resistance patterns. This study aims to empower clinicians in their decision-making and to encourage the development of focused antimicrobial stewardship programs. Such efforts are essential for preserving the efficacy of the limited antibiotics that remain effective against MDR organisms in Saudi Arabia, ultimately improving patient outcomes, mitigating the further spread of resistance, and documenting changes in bacterial resistance patterns.

## Material and methods

### Study design and setting

A retrospective study (January 2020–September 2024) at Prince Sultan Hospital, Western Saudi Arabia, identified regional resistance patterns, which guided antibiotic policies in similar facilities.

### Sample collection and processing

We included different samples, such as blood cultures (BCs), urine (URN) (voided, suprapubic), lower respiratory tract (LRT) samples (sputum, endotracheal tube, tracheal aspirate), and wound swabs (WS) (pus and discharge). We included a total of 5,626 clinical specimens (1,438 BCs, 3,022 URNs, 875 LRTs, and 291 WSs) that yielded significant growth during the study period.

### Bacterial identification and antibiotic susceptibility testing

Our laboratory team identified all organisms using established microbiological techniques. Antibiotic susceptibility testing was performed in accordance with Clinical and Laboratory Standards Institute (CLSI) guidelines. Data were collected on the types of pathogenic organisms, specimen sources, and antibiotic susceptibility patterns.

### Antibiotics tested

These antibiotics were chosen based on their clinical relevance and frequency of use in our setting: ampicillin (AM), penicillin (P), amikacin (AK), amoxicillin (AMOX), amoxicillin/clavulanic acid (AUG), amphotericin B (AMB), ampicillin/sulbactam (A/S), aztreonam (AZT), caspofungin (CAS), cefazolin (CFZ), cefepime (CPM), cephalothin (CF), cefotaxime (CFT), cefoxitin (CFX), ceftazidime (CAZ), ceftazidime/avibactam MIC (CAZ/AVI), ceftolozane/tazobactam (CEF/TAZ), ceftriaxone (CAX), cefuroxime sodium (CRM), chloramphenicol (C), oxacillin (OX), ciprofloxacin (CP), clindamycin (CD), erythromycin (E), fosfomycin (FOS), colistin (CT), gentamicin (GM), levofloxacin (LV), linezolid (LEN), imipenem (IMP), meropenem (MEM), nitrofurantoin (FD), fusidic acid (FA), piperacillin/tazobactam (PI/T), moxifloxacin (MX), rifampin (RIF), ticarcillin/clavulanic acid (TIM), teicoplanin (TP), tetracycline (TE), tigecycline (TGC), tobramycin (TO), trimethoprim/sulfamethoxazole (T/S), sulfamethoxazole/trimethoprim (SXT), polymyxin B (PB), vancomycin (V), minocycline (MN), and norfloxacin (NXN).

### Statistical analysis

Descriptive statistics were used for the antibiogram analysis. We calculated the percentages of resistance to each combination of organisms and antibiotics for each year, then summed the percentages across all years for both men and women. Time-based trends in antimicrobial resistance were evaluated using binary logistic regression models applied separately to each antibiotic, with resistance status coded as a binary outcome (0 = sensitive, 1 = resistant) and year treated as a continuous predictor. Odds ratios per year with corresponding confidence intervals were estimated to assess the direction and magnitude of resistance changes over time. To provide an overall comparison across antibiotics, forest plots were constructed summarizing the odds ratios derived from individual models. In addition, temporal changes in the distribution of bacterial organisms and infection sites were assessed using multinomial logistic regression, given the multi-category nature of these outcomes. Graphical representations were generated to illustrate resistance trends and distributional changes over the study period.

### Ethical considerations

The study was approved by the Institutional Review Board. As this was a retrospective study using anonymized laboratory data, the need for individual patient consent was waived. The authors declare no conflicts of interest related to this study. This research did not receive any specific grant from funding agencies in the public, commercial, or not-for-profit sectors.

## Results

**Figure 1:**
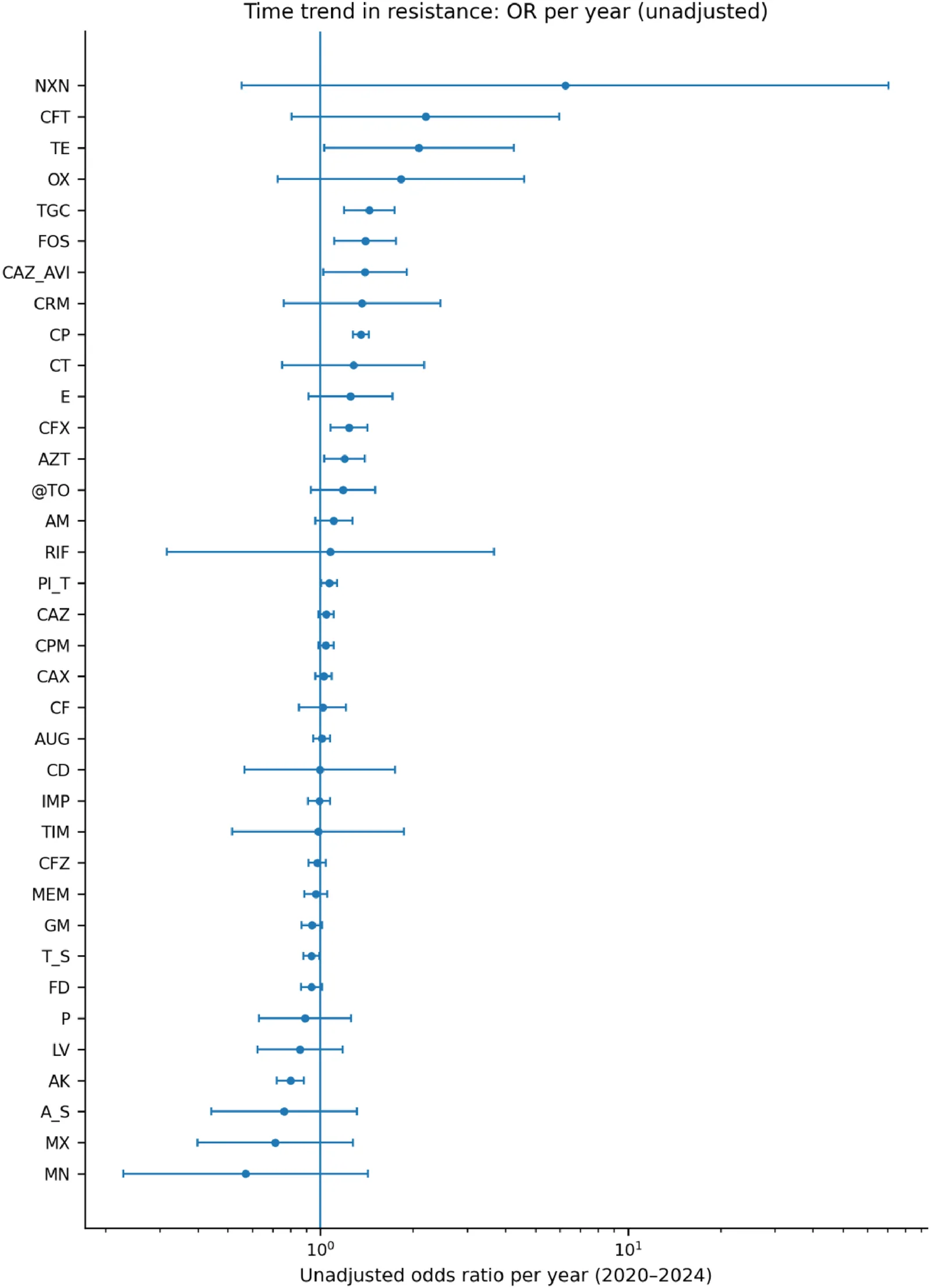
Forest plot summarizing antibiotic-specific temporal resistance trends based on separate logistic regression models ratios per year from 2020 to 2024.

Analysis of antimicrobial resistance trends from 2020 to 2024 revealed marked heterogeneity in time-based patterns across the tested antibiotics, as summarized in the forest plot (**Figure 1**) and the corresponding summary of odds ratios per year (**Table 1**).

**Table 1:** Summary of temporal resistance trends by antibiotic (2020–2024).

Antibiotic (abbreviation)	Antibiotic (full name)	Tested isolates (n)	OR per year	95% CI	P-value	Trend direction
@TO	Tobramycin	296	1.183	0.930–1.505	0.1702	Stable
AK	Amikacin	2,359	0.798	0.721–0.884	<0.001*	Decreasing
AM	Ampicillin	1,285	1.105	0.963–1.268	0.1531	Stable
AUG	Amoxicillin–clavulanic acid	2,054	1.01	0.949–1.074	0.7587	Stable
AZT	Aztreonam	494	1.196	1.027–1.393	0.0212*	Increasing
A_S	Ampicillin–sulbactam	77	0.763	0.442–1.316	0.33	Stable
CAX	Cefuroxime	2,132	1.024	0.963–1.089	0.4484	Stable
CAZ	Ceftazidime	2,383	1.042	0.983–1.106	0.1648	Stable
CAZ_AVI	Ceftazidime–avibactam	101	1.395	1.020–1.909	0.0373*	Increasing
CD	Clindamycin	104	0.994	0.566–1.744	0.9824	Stable
CF	Cephalothin	575	1.016	0.853–1.211	0.8591	Stable
CFT	Ceftriaxone	39	2.196	0.806–5.983	0.1239	Stable
CFX	Cefoxitin	952	1.238	1.079–1.421	0.0023*	Increasing
CFZ	Cefazolin	1,893	0.975	0.912–1.042	0.4524	Stable
CP	Ciprofloxacin	2,449	1.351	1.273–1.434	<0.001*	Increasing
CPM	Cefepime	2,375	1.042	0.982–1.105	0.1742	Stable
CRM	Clarithromycin	257	1.365	0.758–2.458	0.3006	Stable
CT	Colistin	578	1.279	0.752–2.177	0.3639	Stable
E	Erythromycin	105	1.252	0.914–1.714	0.1613	Stable
ESBL	Extended-spectrum β-lactamase phenotype	1,136	0.722	0.650–0.801	<0.001*	Decreasing
FD	Fusidic acid	1,847	0.934	0.863–1.012	0.0941	Stable
FOS	Fosfomycin	1,183	1.397	1.108–1.761	0.0047*	Increasing
GM	Gentamicin	2,443	0.938	0.868–1.014	0.1063	Stable
IMP	Imipenem	2,391	0.99	0.910–1.077	0.8149	Stable
LV	Levofloxacin	124	0.858	0.623–1.182	0.3489	Stable
MEM	Meropenem	2,410	0.966	0.887–1.051	0.4213	Stable
MN	Minocycline	36	0.57	0.228–1.425	0.2295	Stable
MX	Moxifloxacin	55	0.713	0.398–1.277	0.2557	Stable
NXN	Norfloxacin	201	6.25	0.554–70.456	0.1381	Stable
OX	Oxacillin	104	1.827	0.725–4.600	0.201	Stable
P	Penicillin	103	0.89	0.630–1.255	0.5049	Stable
PI_T	Piperacillin–tazobactam	2,360	1.066	1.005–1.131	0.0325*	Increasing
RIF	Rifampicin	75	1.076	0.316–3.667	0.9065	Stable
TE	Tetracycline	82	2.091	1.028–4.255	0.0418*	Increasing
TGC	Tigecycline	1,186	1.442	1.193–1.744	0.0002*	Increasing
TIM	Ticarcillin–clavulanic acid	48	0.982	0.517–1.867	0.9564	Stable
T_S	Trimethoprim–sulfamethoxazole	2,361	0.935	0.881–0.991	0.0239*	Decreasing

On the one hand, antibiotics showed a clear increase in resistance over the years (OR >1 and CIs entirely above 1), as follows: TGC (tigecycline), FOS (fosfomycin), CAZ-AVI (ceftazidime-avibactam), CP (cephalothin), CFX (cefoxitin), and AZT (aztreonam). These antibiotics showed a significant rise in resistance over the five-year study period. On the other hand, some antibiotics with an increasing trend (OR >1, but the CIs cross 1) are as follows: NXN (norfloxacin), CFT (ceftriaxone), TE (tetracycline), OX (oxacillin), CRM (clarithromycin), CT (colistin), E (erythromycin), and RIF (rifampicin). There is a signal toward increasing resistance, with no statistical certainty for this trend. Antibiotics showing a decreasing trend (OR <1) are as follows: P (penicillin), LV (levofloxacin), AK (amikacin), A_S (ampicillin–sulbactam), MX (moxifloxacin), and MN (minocycline). The decreasing trend of these antibiotics showed a pattern but was not statistically confirmed. The remaining antibiotics illustrated a stable trend with no significant changes (OR ≈ 1 and narrow CIs crossing 1).

These contrasting patterns were further evident in the individual time-trend plots, which showed variability in both the magnitude and direction of year-to-year resistance changes among antibiotics (**Supplementary Figure S1**-**S37**). In some cases, resistance proportions increased steadily across consecutive years, whereas in others, fluctuations were observed without a consistent directional trend.

**Figure 2:**
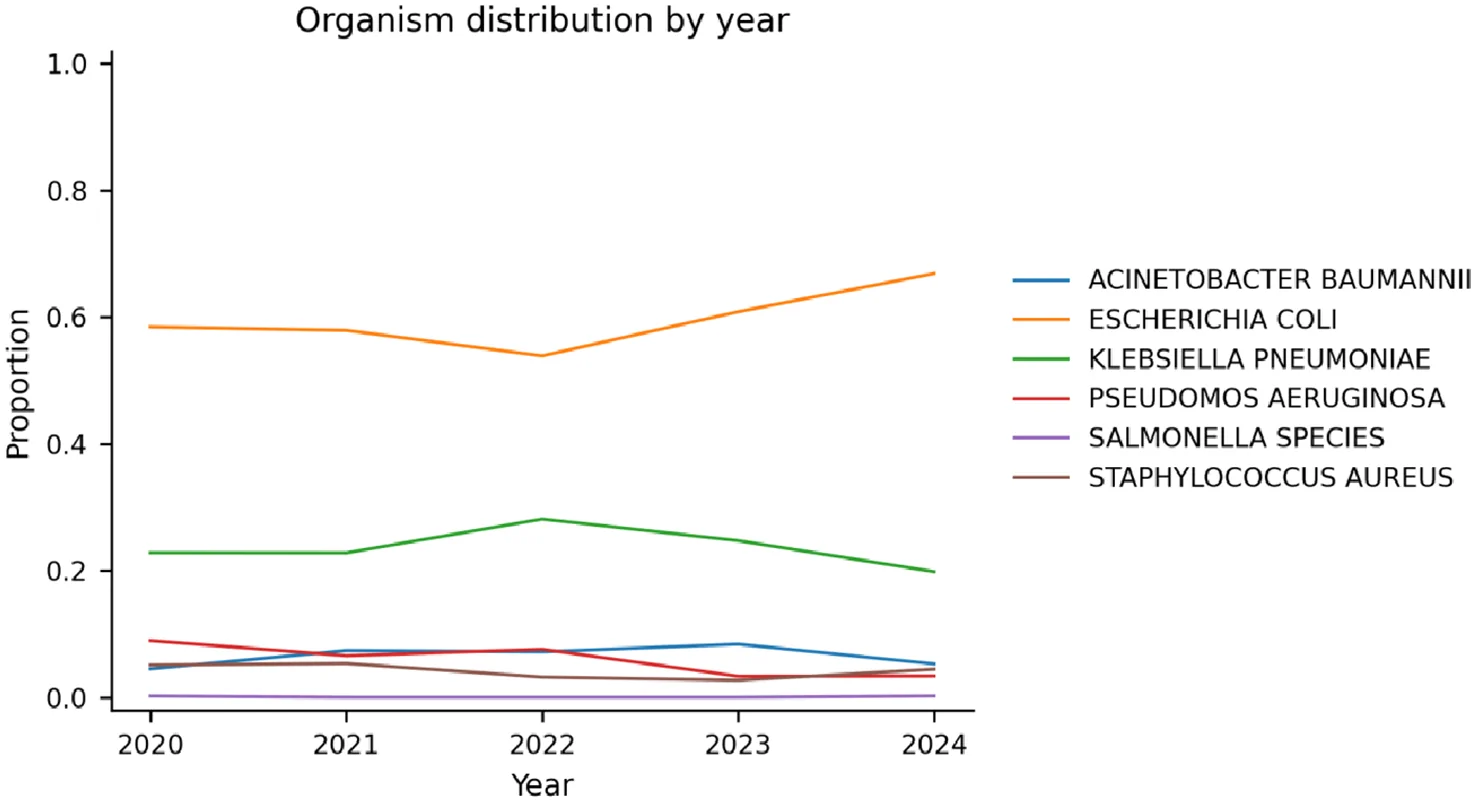
Time-based changes in bacterial organism distribution assessed using multinomial logistic regression.

In parallel with resistance trends, the composition of bacterial organisms contributing to clinical infections changed over time (**Figure 2**; **Table 2**). The figure illustrated that certain species became more prominent during the later years of the study period, particularly *E. coli*. While others demonstrated a decline in relative frequency (*K. pneumoniae, P. aeruginosa* and *S. aureus*), reflecting time-based shifts in the bacterial population structure over a five-year period of five years. Non-duplicate bacterial isolates from community-acquired infections were provided by a clinical diagnostic laboratory. The dataset comprised 1,540 *E. coli*, 614 K*. pneumoniae*, 172 *Acinetobacter* sp., 151 P*. aeruginosa*, and 105 *S. aureus*.

**Table 2:** Distribution of bacterial organisms by study year (%).

Organism	2020	2021	2022	2023	2024
*Acinetobacter baumannii*	4.5	7.4	7.2	8.4	5.3
*Escherichia coli*	58.5	57.9	53.9	60.9	66.9
*Klebsiella pneumoniae*	22.8	22.8	28.1	24.7	19.8
*Pseudomonas aeruginosa*	9.0	6.6	7.6	3.3	3.4
*Salmonella* species	0.2	0.0	0.0	0.0	0.2
*Staphylococcus aureus*	5.1	5.3	3.2	2.7	4.4

**Figure 3:**
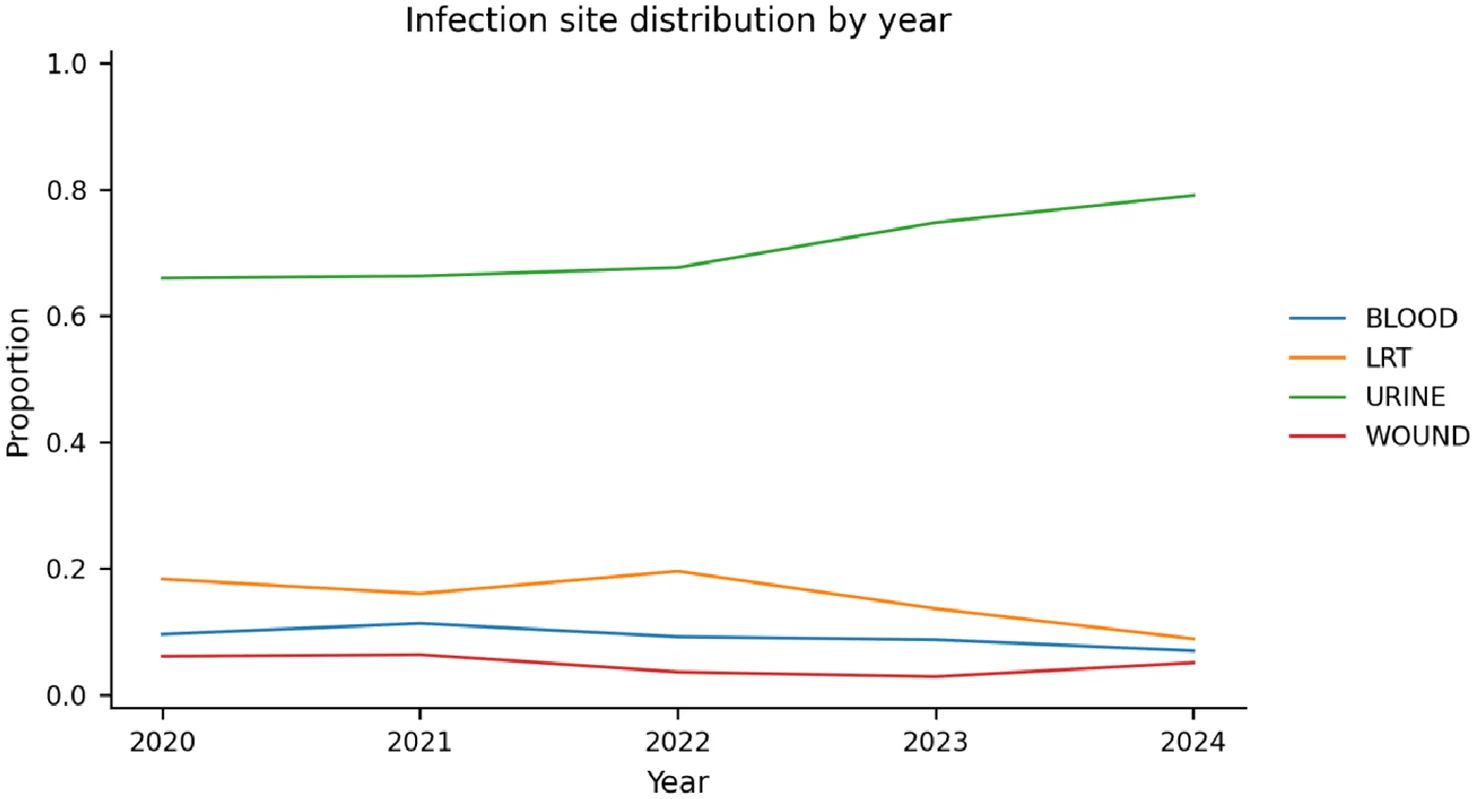
Time-based changes in infection site distribution assessed using multinomial logistic regression.

Similarly, the distribution of infection sites varied across the study years, as illustrated in **Figure 3**; **Table 3**, showing changes in the relative contribution of different specimen sources. The changes were illustrated by an increase in urinary tract infections, a dramatic decrease in lower respiratory tract infections (LRTs), and a slight decrease in blood and wound infections over the five-year period. Collectively, these findings demonstrated that antimicrobial resistance trends occurred alongside concurrent time-based variation in both organism and infection site distributions, underscoring the dynamic nature of the clinical and microbiological landscape over the study period.

**Table 3:** Distribution of infection sites by study year (%).

Infection site	2020	2021	2022	2023	2024
Blood	9.6	11.3	9.2	8.7	7.0
LRT	18.3	16.0	19.6	13.6	8.9
Urine	66.0	66.3	67.7	74.8	79.1
Wound	6.1	6.4	3.6	2.9	5.1

## Discussion

Many pathogenic bacteria were studied in multiple sites, but only those bacteria that showed significant antibiotic resistance were documented in this study. Many antibiotics showed an increasing pattern over the five-year period. We categorized these antibiotics as β-lactams, which are cell wall synthesis inhibitors, and non-β-lactams. The β-lactams illustrated in this study with an increasing pattern were aztreonam (a monobactam used for Gram-negative bacteria), cefoxitin (a second-generation cephalosporin), ceftazidime-avibactam, and piperacillin-tazobactam (β-lactams with β-lactamase inhibitors). These antibiotics are broad-spectrum options, and some are prescribed for the treatment of complicated UTI and sepsis as intravenous (IV) agents and are considered last-line β-lactams with β-lactamase inhibitors (Hidalgo et al., 2016). The IV broad spectrum aztreonam and piperacillin-tazobactam are often used for the treatment of UTI, specifically in patients with severe β-lactam allergy (Kaye et al., 2022).

### Distribution of ESBL-producing isolates

The study investigated 2,033 isolates, identifying that 29% (n = 607) were ESBL producers. In this ESBL context, a reserve agent used is ceftazidime-avibactam (Tamma et al., 2024). The particular concern is antibiotics often used for MDR infections, such as tigecycline resistance (Park et al., 2020). These drugs may be losing reliability and should prompt reevaluation of empirical use (Kadri et al., 2018).

Antibiotics in the current study that demonstrated an increasing but non-significant trend were as follows: norfloxacin, tetracycline, rifampicin, β-lactams (ceftriaxone, oxacillin), clarithromycin, erythromycin, and colistin. Resistance to these antibiotics may need continued surveillance, but there is no recent classification indicating an increase in resistance. On the other hand, the resistance patterns of many carbapenems and aminoglycosides stayed the same over time. Therefore, the emergence of resistance in the antibiotics showing an increasing trend suggests therapeutic exhausting. This has been reported globally with a similar trend (Reynolds et al., 2022).

Resistance patterns differed across species and infection sites, underscoring the importance of developing and implementing up-to-date, site-specific treatment guidelines and pathogen-targeted therapeutic strategies. The current study indicates the potential spread of carbapenems-producing *E. coli*. *E. coli* showed fluctuations in total numbers over the five years; overall ESBL rates have been increasing over this time interval. This aligns with the global literature reports particularly on urinary tract infections over the last decade (Cantas et al., 2016). The prevalence in some regions has increased from 44% to 59% over the last 10 years (Mączyńska et al., 2023).

### Escalating resistance to “last resort” antibiotics

It is especially concerning that tigecycline and ceftazidime-avibactam are becoming less effective, since these are often used to treat *Enterobacterales* that are resistant to carbapenems (CRE). These agents are frequently considered “last-line” therapies against carbapenem-resistant Enterobacterales (CRE) (European Centre for Disease Prevention and Control (ECDC), 2025). Results from the current study indicate a statistically significant increasing trend for both antibiotics, with tigecycline exhibiting an odds ratio (OR) of 1.442 (p = 0.0002) and ceftazidime-avibactam demonstrating an OR of 1.395 (p = 0.0373). Global studies indicate that this phenomenon may be influenced by the heightened use of these agents during the COVID-19 pandemic and the ensuing selective pressure (U. S. Centers for Disease Control and Prevention (CDC), 2022).

### Impact of COVID-19 and selective pressure

The time frame of the current study (2020–2024) and its correlation with the COVID-19 outbreak suggest a relationship between pandemic-associated factors and the development of antibiotic resistance. This is believed to have contributed to the development of resistance to several antibiotic agents (Wang et al., 2026). During the early waves of the outbreak, a large proportion of patients were administered broad-spectrum antibiotics empirically, despite there being no confirmed cases of bacterial co-infection. As a result, strong selective pressure was exerted, resulting in the proliferation of resistant strains.

It has been recently established by medical researchers that resistance to tigecycline and ceftazidime-avibactam may develop within one month under the selective pressure induced by β-lactam and TGC treatment. Non-susceptibility to tigecycline in some clinical settings increased from virtually 0% in 2019 to 71% in 2022, whereas resistance to CAZ-AVI increased from 27% to 70%. This may be attributed to the reallocation of healthcare resources and the suspension of antimicrobial stewardships (ASPs) due to the focus on addressing the consequences of the COVID-19 outbreak (Han et al., 2021).

### Stability and declining resistance trends

Although an increase occurred in some regions, many antibiotics had stable or even declining resistance trends. There were stable trends for most common antibiotics, such as the carbapenems: imipenem (IMP) and meropenem (MEM), with no significant increases each year (OR ≈1). Resistance trends showed a significant decline for amikacin (AK) (OR 0.798, p <0.001) and trimethoprim-sulfamethoxazole (T_S) (OR 0.935, p = 0.0239). According to regional surveillance studies conducted in Saudi Arabia and China, amikacin shows very high susceptibility, sometimes below 5%, while resistance to drugs such as ceftriaxone and fluoroquinolones is increasing (Khalid et al., 2025).

In conclusion, this study highlights a pivotal transformation in the epidemiological framework of antimicrobial resistance (AMR), marked by the rising incidence of extended-spectrum β-lactamase (ESBL)-producing *E. coli* and a notable increase in resistance to “last-resort” antibiotics. According to the study findings, traditional classes of drugs, such as aminoglycosides and carbapenems, have remained relatively stable. However, resistance to newer agents, such as ceftazidime-avibactam and tigecycline, has increased rapidly. Such trends, particularly the year-on-year increases in resistance to ceftazidime-avibactam (OR 1.395) and tigecycline (OR 1.442), indicate an emerging state of “therapeutic exhaustion,” similar to the global trend of declining treatment alternatives for carbapenem-resistant Enterobacterales (CRE).

It becomes evident that the emergence of antibiotic resistance is linked to the COVID-19 pandemic through the selective pressure caused by the overprescription of broad-spectrum antibiotics (U. S. Centers for Disease Control and Prevention (CDC), 2022). As increasing resistance rates are observed among last-line antimicrobial agents, it becomes necessary to reconsider current empiric treatment approaches. The use of these agents without prior confirmation of susceptibility may become untenable, underscoring the need to revitalize ASPs to ensure the continued effectiveness of the existing antibiotic arsenal. An interesting variation in the population structure of the studied bacteria was identified in the current study, specifically an increase in the proportion of *E. coli* isolated from UTIs, whereas other strains, such as *K. pneumoniae*, were less represented among the collected samples. This information should be considered during the development of strategies for future studies and treatment in the region. Increasing resistance to antibiotics prescribed to treat complex infections represents a potential threat to public health. It is essential that this shifting disease burden be considered when planning for future regional testing and treatment programs. The emergence of drug resistance among these infections is a public health concern. This study represents a significant first step toward local and regional monitoring of such diseases. It emphasizes that, without strict intervention and careful antibiotic use, the medical community may soon have to deal with infections for which no viable therapeutic options exist.

## Data Availability

The original contributions presented in the study are included in the article/**Supplementary Material**. Further inquiries can be directed to the corresponding authors.

## Supplementary material

Supplementary Figure S1 Amikacin resistance time-trend analysis derived from an individual binary logistic regression model (2020–2024).

Supplementary Figure S2 Ampicillin resistance time-trend analysis derived from an individual binary logistic regression model (2020–2024).

Supplementary Figure S3 Amoxicillin–clavulanic acid resistance time-trend analysis derived from an individual binary logistic regression model (2020–2024).

Supplementary Figure S4 Cefuroxime resistance time-trend analysis derived from an individual binary logistic regression model (2020–2024).

Supplementary Figure S5 Ceftazidime resistance time-trend analysis derived from an individual binary logistic regression model (2020–2024).

Supplementary Figure S6 Ceftriaxone resistance time-trend analysis derived from an individual binary logistic regression model (2020–2024).

Supplementary Figure S7 Cefazolin resistance time-trend analysis derived from an individual binary logistic regression model (2020–2024).

Supplementary Figure S8 Cephalothin resistance time-trend analysis derived from an individual binary logistic regression model (2020–2024).

Supplementary Figure S9 Cefepime resistance time-trend analysis derived from an individual binary logistic regression model (2020–2024).

Supplementary Figure S10 Extended-spectrum β-lactamase phenotype resistance time-trend analysis derived from an individual binary logistic regression model (2020–2024).

Supplementary Figure S11 Gentamicin resistance time-trend analysis derived from an individual binary logistic regression model (2020–2024).

Supplementary Figure S12 Meropenem resistance time-trend analysis derived from an individual binary logistic regression model (2020–2024).

Supplementary Figure S13 Trimethoprim–sulfamethoxazole resistance time-trend analysis derived from an individual binary logistic regression model (2020–2024).

Supplementary Figure S14 Tigecycline resistance time-trend analysis derived from an individual binary logistic regression model (2020–2024).

Supplementary Figure S15 Ceftazidime–avibactam resistance time-trend analysis derived from an individual binary logistic regression model (2020–2024).

Supplementary Figure S16 Cephalothin resistance time-trend analysis derived from an individual binary logistic regression model (2020–2024).

Supplementary Figure S17 Cefoxitin resistance time-trend analysis derived from an individual binary logistic regression model (2020–2024).

Supplementary Figure S18 Imipenem resistance time-trend analysis derived from an individual binary logistic regression model (2020–2024).

Supplementary Figure S19 Piperacillin–tazobactam resistance time-trend analysis derived from an individual binary logistic regression model (2020–2024).

Supplementary Figure S20 Ampicillin–sulbactam resistance time-trend analysis derived from an individual binary logistic regression model (2020–2024).

Supplementary Figure S21 Aztreonam resistance time-trend analysis derived from an individual binary logistic regression model (2020–2024).

Supplementary Figure S22 Colistin resistance time-trend analysis derived from an individual binary logistic regression model (2020–2024).

Supplementary Figure S23 Fusidic acid resistance time-trend analysis derived from an individual binary logistic regression model (2020–2024).

Supplementary Figure S24 Fosfomycin resistance time-trend analysis derived from an individual binary logistic regression model (2020–2024).

Supplementary Figure S25 Clindamycin resistance time-trend analysis derived from an individual binary logistic regression model (2020–2024).

Supplementary Figure S26 Erythromycin resistance time-trend analysis derived from an individual binary logistic regression model (2020–2024).

Supplementary Figure S27 Levofloxacin resistance time-trend analysis derived from an individual binary logistic regression model (2020–2024).

Supplementary Figure S28 Moxifloxacin resistance time-trend analysis derived from an individual binary logistic regression model (2020–2024).

Supplementary Figure S29 Oxacillin resistance time-trend analysis derived from an individual binary logistic regression model (2020–2024).

Supplementary Figure S30 Penicillin resistance time-trend analysis derived from an individual binary logistic regression model (2020–2024).

Supplementary Figure S31 Rifampicin resistance time-trend analysis derived from an individual binary logistic regression model (2020–2024).

Supplementary Figure S32 Tetracycline resistance time-trend analysis derived from an individual binary logistic regression model (2020–2024).

Supplementary Figure S33 Clarithromycin resistance time-trend analysis derived from an individual binary logistic regression model (2020–2024).

Supplementary Figure S34 Norfloxacin resistance time-trend analysis derived from an individual binary logistic regression model (2020–2024).

Supplementary Figure S35 Tobramycin resistance time-trend analysis derived from an individual binary logistic regression model (2020–2024).

Supplementary Figure S36 Minocycline resistance time-trend analysis derived from an individual binary logistic regression model (2020–2024).

Supplementary Figure S37 Ticarcillin–clavulanic acid resistance time-trend analysis derived from an individual binary logistic regression model (2020–2024).
