# Supplementary material for: Antibiogram of pathogenic bacterial isolates from various samples in Prince Sultan Hospital, Western Region, Saudi Arabia. Retrospective study from 2020 to 2024
Source: Front Antibiot. 2026 Aug 12;5:1872911. doi: 10.3389/frabi.2026.1872911 (PMC13506799; doi:10.3389/frabi.2026.1872911)
Supplement: Supplementary file 1 [file DataSheet1.docx]

**Figure S1.** Amikacin resistance time-trend analysis derived from an individual binary logistic regression model (2020–2024).

**Figure S2.** Ampicillin resistance time-trend analysis derived from an individual binary logistic regression model (2020–2024).

**Figure S3.** Amoxicillin–clavulanic acid resistance time-trend analysis derived from an individual binary logistic regression model (2020–2024).

**Figure S4.** Cefuroxime resistance time-trend analysis derived from an individual binary logistic regression model (2020–2024).

**Figure S5.** Ceftazidime resistance time-trend analysis derived from an individual binary logistic regression model (2020–2024).

**Figure S6.** Ceftriaxone resistance time-trend analysis derived from an individual binary logistic regression model (2020–2024).

**Figure S7.** Cefazolin resistance time-trend analysis derived from an individual binary logistic regression model (2020–2024).

**Figure S8**. Cephalothin resistance time-trend analysis derived from an individual binary logistic regression model (2020–2024).

**Figure S9.** Cefepime resistance time-trend analysis derived from an individual binary logistic regression model (2020–2024).

**Figure S10.** Extended-spectrum β-lactamase phenotype resistance time-trend analysis derived from an individual binary logistic regression model (2020–2024).

**Figure S11.** Gentamicin resistance time-trend analysis derived from an individual binary logistic regression model (2020–2024).

**Figure S12.** Meropenem resistance time-trend analysis derived from an individual binary logistic regression model (2020–2024).

**Figure S13.** Trimethoprim–sulfamethoxazole resistance time-trend analysis derived from an individual binary logistic regression model (2020–2024).

**Figure S14.** Tigecycline resistance time-trend analysis derived from an individual binary logistic regression model (2020–2024).

**Figure S15.** Ceftazidime–avibactam resistance time-trend analysis derived from an individual binary logistic regression model (2020–2024).

**Figure S16**. Cephalothin resistance time-trend analysis derived from an individual binary logistic regression model (2020–2024).

**Figure S17.** Cefoxitin resistance time-trend analysis derived from an individual binary logistic regression model (2020–2024).

**Figure S18.** Imipenem resistance time-trend analysis derived from an individual binary logistic regression model (2020–2024).

**Figure S19.** Piperacillin–tazobactam resistance time-trend analysis derived from an individual binary logistic regression model (2020–2024).

**Figure S20.** Ampicillin–sulbactam resistance time-trend analysis derived from an individual binary logistic regression model (2020–2024).

**Figure S21.** Aztreonam resistance time-trend analysis derived from an individual binary logistic regression model (2020–2024).

**Figure S22.** Colistin resistance time-trend analysis derived from an individual binary logistic regression model (2020–2024).

**Figure S23.** Fusidic acid resistance time-trend analysis derived from an individual binary logistic regression model (2020–2024).

**Figure S24.** Fosfomycin resistance time-trend analysis derived from an individual binary logistic regression model (2020–2024).

**Figure S25.** Clindamycin resistance time-trend analysis derived from an individual binary logistic regression model (2020–2024).

**Figure S26.** Erythromycin resistance time-trend analysis derived from an individual binary logistic regression model (2020–2024).

**Figure S27.** Levofloxacin resistance time-trend analysis derived from an individual binary logistic regression model (2020–2024).

**Figure S28.** Moxifloxacin resistance time-trend analysis derived from an individual binary logistic regression model (2020–2024).

**Figure S29.** Oxacillin resistance time-trend analysis derived from an individual binary logistic regression model (2020–2024).

**Figure S30.** Penicillin resistance time-trend analysis derived from an individual binary logistic regression model (2020–2024).

**Figure S31.** Rifampicin resistance time-trend analysis derived from an individual binary logistic regression model (2020–2024).

**Figure S32.** Tetracycline resistance time-trend analysis derived from an individual binary logistic regression model (2020–2024).

**Figure S33.** Clarithromycin resistance time-trend analysis derived from an individual binary logistic regression model (2020–2024).

**Figure S34.** Norfloxacin resistance time-trend analysis derived from an individual binary logistic regression model (2020–2024).

**Figure S35.** Tobramycin resistance time-trend analysis derived from an individual binary logistic regression model (2020–2024).

**Figure S36.** Minocycline resistance time-trend analysis derived from an individual binary logistic regression model (2020–2024).

**Figure S37.** Ticarcillin–clavulanic acid resistance time-trend analysis derived from an individual binary logistic regression model (2020–2024).
